# Imagine a world without cancer

**DOI:** 10.1186/1471-2407-14-186

**Published:** 2014-03-14

**Authors:** Björn LDM Brücher, Gary Lyman, Richard van Hillegersberg, Raphael E Pollock, Florian Lordick, Han-Kwang Yang, Toshikazu Ushijima, Khay-Guan Yeoh, Tomas Skricka, Wojciech Polkowski, Grzegorz Wallner, Vic Verwaal, Alfredo Garofalo, Domenico D’Ugo, Franco Roviello, Hans-Ulrich Steinau, Timothy J Wallace, Martin Daumer, Nitah Maihle, Thomas J Reid, Michel Ducreux, Yuko Kitagawa, Alexander Knuth, Bruno Zilberstein, Scott R Steele, Ijaz S Jamall

**Affiliations:** 1 Theodor-Billroth-Academy®, Munich, Germany; 2 Theodor-Billroth-Academy®, Sacramento, CA, USA; 3 Theodor-Billroth-Academy®, Richmond, VA, USA; 4 INCORE, International Consortium of Research Excellence of the Theodor- Billroth-Academy®, Munich, Germany; 5 INCORE, International Consortium of Research Excellence of the Theodor- Billroth-Academy®, Sacramento, CA, USA; 6 INCORE, International Consortium of Research Excellence of the Theodor- Billroth-Academy®, Richmond, VA, USA; 7 Bon Secours Cancer Institute, Richmond, VA, USA; 8 Fred Hutchinson Cancer Research Center, University of Washington, Seattle, WA, USA; 9 Department of Surgery, University Medical Center, Utrecht, The Netherlands; 10 Surgical Oncology, Ohio State University Comprehensive Cancer Center, Columbus, OH, USA; 11 Medical Oncology, University of Leipzig, Leipzig, Germany; 12 Department of Surgery, Seoul National University Hospital, Seoul, Korea; 13 Division of Epigenomics, National Cancer Center Research Institute, Tokyo, Japan; 14 Yong Loo Lin School of Medicine, National University of Singapore, Singapore, Singapore; 15 Department of Surgery, Masaryk University, Brno, Czech Republic; 16 Surgery, Medical University of Lublin, Lublin, Poland; 17 The Netherlands Cancer Institute-Antoni van Leeuwenhoek Hospital, Amsterdam, The Netherlands; 18 Institutio Nazionale Tumori Regina Elena, Roma, Italy; 19 Department of Surgery, Catholic University Rome A, Rome, Italy; 20 Surgical Oncology, University of Siena, Siena, Italy; 21 University of Essen, Essen, Germany; 22 Sylvia Lawry Center for MS Research, Munich, Germany; 23 Biochemistry and Molecular Biology, Georgia Regents University, Augusta, GA, USA; 24 Regional Cancer Center Memorial Hospital of South Bend, South Bend, IN, USA; 25 Departement of Medicine, Institut Gustave Roussy, Villejuif, Universite Paris-Sud, Le Kremlin, Bicetre, France; 26 Department of Surgery, Keio University, Tokyo, Japan; 27 National Center for Cancer Care and Research, Doha, Qatar; 28 Digestive Surgery Division, Sao Paulo University, San Paulo, Brazil; 29 Department of Surgery, Madigan Army Medical Center, Tacoma, WA, USA; 30 Risk-based Decisions Inc, Sacramento, CA, USA

**Keywords:** Cancer, Carcinogenesis, Multimodal therapy, Cancer classification, Personalized anticancer therapy, Individualized anticancer therapy

## Abstract

**Background:**

Since the “War on Cancer” was declared in 1971, the United States alone has expended some $300 billion on research, with a heavy focus on the role of genomics in anticancer therapy. Voluminous data have been collected and analyzed. However, in hindsight, any achievements made have not been realized in clinical practice in terms of overall survival or quality of life extended. This might be justified because cancer is not one disease but a conglomeration of multiple diseases, with widespread heterogeneity even within a single tumor type.

**Discussion:**

Only a few types of cancer have been described that are associated with one major signaling pathway. This enabled the initial successful deployment of targeted therapy for such cancers. However, soon after this targeted approach was initiated, it was subverted as cancer cells *learned and reacted* to the initial treatments, oftentimes rendering the treatment less effective or even completely ineffective. During the past 30 plus years, the cancer classification used had, as its primary aim, the facilitation of communication and the exchange of information amongst those caring for cancer patients with the end goal of establishing a standardized approach for the diagnosis and treatment of cancers. This approach should be modified based on the recent research to affect a change from a service-based to an outcome-based approach. The vision of achieving long-term control and/or eradicating or curing cancer is far from being realized, but not impossible. In order to meet the challenges in getting there, any newly proposed anticancer strategy must integrate a personalized treatment outcome approach. This concept is predicated on tumor- and patient-associated variables, combined with an individualized response assessment strategy for therapy modification as suggested by the patient’s own results. As combined strategies may be outcome-orientated and integrate tumor-, patient- as well as cancer-preventive variables, this approach is likely to result in an optimized anticancer strategy.

**Summary:**

Herein, we introduce such an anticancer strategy for all cancer patients, experts, and organizations: *Imagine a World without Cancer*.

## Background

**Figure 1:**
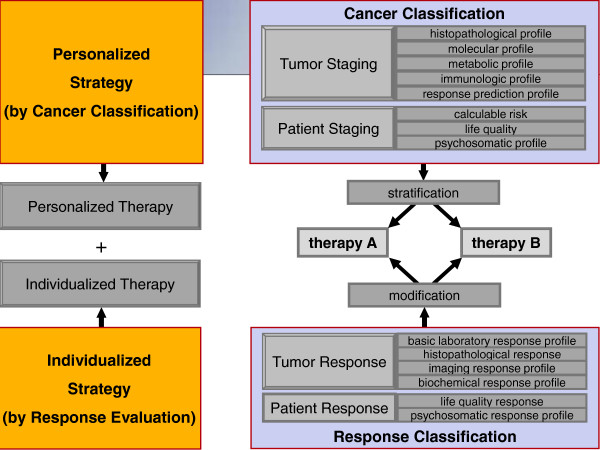
**Figure Combined strategy with a tumor- *and *** **patient-orientated predictive cancer-staging system with the stratification of different forms of *personalized *** **therapy and the development of a standardized multivariable response evaluation system with the consequence of future standardized therapy modifications (*individualized *** **cancer strategy).**

Recently, Kohane stated that *“the size and complexity of needed multidimensional characterization of patients will lead to far more complex diagnostic and prognostic categories than are currently in use*” [1]. The end result of this concept is an improved ability to understand interactions between macro-and micro- tumor environments. Ultimately, this may allow us to precisely characterize patients by more components, dig deeper and classify each patient by variations of the individual organ, gene, and even, molecular make-up [1]. Applying this to cancer, the vision of eradicating and/or curing cancer is far from being realized. Meeting this goal in the future, however, requires an approach that should not be focused solely on genotyping. Rather, the future of oncology will have to integrate the increasing power of information achieved by research into rapidly adaptable anticancer treatment strategies. *Imagine a World without Cancer* is clearly a goal of all cancer patients, experts and organizations, and this requires a focused anticancer strategy that culminates in a personalized treatment strategy based on a new cancer classification scheme combined with an assessment of the individualized patient response for therapy modification (Figure 1). Combining both strategies should result in a disease-focused, outcomes-based approach that integrates tumor- and patient-specific variables, where both basic and clinical research results are united in a synergistic, or optimally, in a potentiated manner.

## Discussion

### Where we are - current status of war on cancer

The oldest case of a metastatic cancer case was found in the skeleton of a 2,700 year old male in Siberia, Russia [2] while and the oldest cancer description (1,500 BCE) was discovered in Egypt within the so-called Edwin Smith Papyrus [3,4]. The term cancer is derived from the Greek word for crab, Karkinos, coined by Hippocrates (460–370 BC). Cancer stands for a malignant tumor, one that is characterized by anaplasia (i.e., the loss of normal appearance of cells) and autonomy (loss of inhibition of growth) displaying invasive and tissue-destroying properties [5]. Approximately 130 years ago, a Russian student in the Veterinary Department of the Medical Surgery Academy in St. Petersburg, Mistislas Aleksandrovich Novinsky (1841–1914), obtained a dog with a carcinoma of the nose and transplanted small fragments subcutaneously into a healthy puppy, thereby demonstrating growth of the transplanted tumor mass [5,6].

The War on Cancer was declared by President Nixon in1971 when he signed the National Cancer Act, with the primary aim of strengthening the National Cancer Institute (NCI). Since then, the US alone has expended billions on huge data collection and analyses, understanding genomic techniques, and on anticancer treatments. From a “birds-eye” view, it may appear that we have made little progress in eradicating cancer and that outcomes are largely based on the merits, or lack thereof, of any selected therapy. There is a reliance almost exclusively on those therapies that demonstrate statistical significance without carefully evaluating if the patient’s quality of life has improved or whether the overall survival represents a meaningful period - not just simply calculating survival in weeks or months. Thus, we may have relied too heavily on the worship of false gods, and not selected our metrics properly.

Since Novinsky’s experiment in 1876, cancer has been classified, structured, and sub-grouped the tissues of origin (organ-based cancer classification) by tumor size (T), by lymph node involvement (N), and spread to organs (M). This TNM classification of Malignant Tumors (TNM) has been used by both the International Union Against Cancer (UICC) and the American Joint Committee on Cancer (AJCC) and is presented in the 7th edition of the UICC and AJCC Cancer Staging Manual [7], where it forms the basis for treating cancer patients. Problems do exist: unnecessary invasive diagnostic tests (angiography, diagnostic laparoscopy/laparotomy, mammograms, prostate biopsies, etc.), over-treatment (radiation exposure), and vast over-utilization of medical resources occur; and yet, many still experience misdiagnosis or, at best, a penurious extension in life (though not necessarily an improved quality of life in the extended survival). Improvements need to occur, and soon, but are easier said than done.

Pathologic staging can have mistakes: tissue can be sampled incorrectly or in inadequate amounts relative to the likely tumor mass by different variables: the person who maintains the biopsy/specimen, the investigator, or the evaluation of the pathologist. If cancerous cells are not present within the slide or image, an absence of cancer may be concluded with serious or even fatal consequences for the patient. Additionally, not identifying small numbers of cancerous cells intermixed with healthy cells on one slide can also occur. Moreover, some types of cancer (i.e., brain and spinal cord) are graded by matching the cell type, while others such as blood, bone marrow and lymphomas are classified by phenotype or a combination of location and symptoms (i.e., Ann Arbor classification of lymphomas from 1971) [8]. Yet this results largely in broad classifications of tumors, which to the best of our current understanding may act quite differently on a biological level. An attempt to account for the biological behavior and prognostic relevance of certain tumors is reflected by grade and bulk as introduced by the Cotswold modification [9].

Another undefined point is the number of biopsy samples that optimally should be collected for making a diagnosis with a high probability of being correct. It has been shown that biopsies that are not guided by a 3-D imaging approach of a suspect lesion have odds of detection that are far lower than a clinician might expect [10]. For example, if a cancer is limited to ~5% in all three dimensions and the tissue or organ volume is considered, the odds of finding the cancer through standard biopsy techniques are less than 50%. Translating this to a run-of-the-mill prostate carcinoma, the number of tissue samples would have to be approximately 18–20 randomly collected biopsy specimens. When compared to the usual > 8 biopsies most patients receive, this could increase the odds of finding a potential cancerous lesion to 90-95%.

### Cancer is not a single disease

Although often used in the singular, cancer is not one disease, but rather a collection of more than 100 diseases with some traits in common [11]. There are but a few cases of cancer that have been associated with important signaling pathway (e.g. Bcr-Abl tyrosine kinase in chronic myelogenous leukemia, CML), which has allowed for the deployment of targeted therapy [12]. However, we soon learned, empirically, that this targeted therapeutic approach was subverted over time as the cancer cells *learned and reacted* to the initial treatment, oftentimes rendering the treatment less effective or completely ineffective. As one example, consider Bcr-Abl and its role in CML. Point mutations exist on at least 13 different amino acids distributed over the Abl-kinase domain, which make it difficult to overcome drug resistance. However, once that threshold of drug resistance is reached, the patient usually succumbs to the cancer as the therapy is rendered ineffective [12,13].

It is important to understand that the existing cancer classification scheme was originally intended to facilitate communication and exchange information among physicians and oncologists. This was necessary for both a widely accepted cancer classification system and for standardizing anticancer therapy over the past half century. The change in cancer incidences as well as the achievements have been pointed out recently within the Blueprint of the American Society of Clinical Oncology (ASCO): “*Two out of three people live at least five years after cancer diagnosis, up from roughly one out of two in the 1970s*” and “*The nation’s cancer death rate has dropped 18 percent since the early 1990s, reversing decades of increases*” [14,15]. Although cancer survival rates have improved over the past several decades [16], this can be attributed to larger gains in some cancers including breast, colon, and Hodgkin’s lymphoma, while improvements in survival may (at best) be of barely a few weeks for cancers of the lung, brain, or pancreas [17]. We would argue that many of the gains in cancer therapy have come at the cost of quality of life in cancer survivors, a fact not often incorporated in the statistics. Perhaps by shifting our focus from the killing of cancer cells to merely delaying the ability of cancers to cause illness might yield better results both in prognosis and, importantly, in quality of life extended. This can occur through efforts such as examining the basic biochemical lesions associated with cancer.

### The necessity of including patient- and tumor-associated variables to a modified cancer classification

Will the recent developments in molecular science and, where feasible, surgery to remove the cancer mass cure cancer alone? Hanahan & Weinberg focused on the six biological processes “*during the multi-step development of human tumors: sustaining proliferative signaling, evading growth suppressors, resisting cell death, enabling replicative immortality, inducing angiogenesis, and activation invasion, and metastasis*” [18]. On the other hand, Vogelstein and Kinzler [19] proposed that cancer is a disease that is the result of damage to the DNA with consecutive genetic mutations. Therefore, it has been proposed to include molecular information into traditional classifications such as in many solid cancers. Unfortunately, this information is not yet fully understood and not routinely applied in clinical practice due to the absence of standardized criteria and/or research funding [20].

The inclusion of findings in Genomics/Epigenomics, Metabolomics, Proteomics, Inflammation, Immunology, Adult Stem Cell research as well as response information to the traditional histopathological staging and to patient-associated variables (such as risk calculation profiles, life quality and psychosomatic/spiritual profiling) seems not only important, but also necessary. Jones and Baylin revealed that genetics and epigenetics cooperate at all stages of carcinogenesis, and “*that epigenetic abnormalities in cancer comprise a multitude of aberrations in virtually every component of chromatin involved in packaging the human genome*” [21]. One molecular approach or signaling pathway will not determine the future needs of a cancer therapy that is biologically-oriented and classified, and dictates both treatment and prevention.

Several examples currently exist of an attempt to bridge the molecular changes with treatment, prognosis and outcome. Investigating the TGF-beta1signal transduction revealed that the amplification of the ski-gene, a repressor of TGF-beta1, correlates with significantly worse survival outcomes in colon carcinoma [22]. Moreover deletions in smad4 and smad7 correspond with worse prognosis in chemotherapy-treated colon carcinoma [23,24]. Adding Cetuximab (Erbitux; ImClone Systems Inc.), a monoclonal antibody and inhibitor of EGFR, to multimodal treatment increased efficiency of anticancer therapy in patients with k-RAS wild type non-resectable liver metastasis originally from colorectal carcinoma (CRC) [25]. The complexity of molecular profiling can be seen by the example of adding predictive response information by genotype-wide single nucleotide polymorphisms (SNPs) screening. It was shown that SNPs in patients homozygous for the wild type alleles of LIFR rs3729740 undergoing treatment with Cetuximab (Erbitux; ImClone Systems Inc.) and possibly ANXA11 rs1049550 in those patients receiving Bevacizumab (Avastin; Genentech Inc.) can serve as markers for chemosensitivity to multimodal treatment [26]. Additionally, this was also shown in a recent study in the setting of metastatic colorectal cancer [27]. The increase in molecular profiling through metabolic phenotyping will likely generate patient-specific information on tumor biology, allowing physicians to improve diagnostic capabilities and helping to select the appropriate optimized treatment for that individual tumor in that particular patient [28]. Further categorization has been demonstrated in bladder cancer research, where adding epigenetic mechanisms such as DNA-methylation, histone-modification and ncRNA-expression may result in future potential biomarkers and/or therapeutic targets [29].

In the field of cancer metabolics, the *Warburg* effect, defined by an increased utilization of glucose via glycolysis, is a common biochemical characteristic of cancerous cells [30]. Proliferating cells have intrinsic increased metabolic activities compared to non-proliferating cells [31]. These findings have been clinically addressed earlier, by differentiating cancer patients into metabolic responders and non-responders [32]. An adverse regulator of the Warburg effect, suppressing tumor growth *in vivo,* has also been recently reported [33]. By preventing or slowing the loss of contact inhibition, whereby cancer cells become aware of their neighboring cells and do not run rampant across the geography of a tissue or organ, we can decrease the onset or progression of pathology, organ dysfunction, disease, and death. This approach would support a strategy to develop methods for further analyzing the signaling networks that underlie cancer development, progression, and drug resistance [34]. Another important field in basic cancer research with future impact in personalized oncology is investigating proteins on their structure and function or proteomics. The potential achievements of the Human Proteome Project, a collaboration investigating the complete individual protein information, were reviewed recently [35]. The primary aim of this work is to enable a better understanding of how cancer cells thrive in their environment [35,36]. Future contributing results might be expected on serum-based algorithms stratifying anticancer therapy [37].

Different types of inflammation can have also an effect on initiation, promotion or progression of tumor development [38]. As the function of the stroma in the tumor microenvironment has been underestimated for many years, it has been reported that stromal cell-related cytokines of inflammation such as tumor necrosis factor alpha (TNF-α) activate nuclear factor kappa-light-chain-enhancer of activated B cells (NF-κB) play important but as yet unquantified roles in tumor development [39,40]. For example, suppression of NF-κB by anti-TNF-α leads to an inhibition of disease progression in hepatocellular carcinoma [40]. The association of tumor-infiltrating T-cells and their well-defined correlation with clinical outcomes in ovarian, breast, prostate, renal, esophageal, colorectal carcinomas, and melanomas were cardinal observations in the understanding of the role that immunology plays in cancer [41-47]. Inflammation, as well as the tumor environment, interacts in all different types of immune cells [38]. The clinical findings of immuno-oncology in prostate cancer and melanoma have resulted in significant contributions in the struggle against cancer with the least adverse effects of any known cancer therapy currently in use [48,49].

The Nobel-Prize winning discovery of pluripotency, demonstrated by Shinya Yamanaka in 2006, wherein a differentiated cell altered into an induced pluripotent stem cell by a defined set of transcription factors has opened up another potentially lucrative therapeutic approach against cancer [50,51]. All these approaches viewed collectively and applied judiciously can prevent the known obstacles of drug resistance, evolution of alternate paths of tumorigenesis, cytotoxicity and, importantly, improve the quality of life for cancer patients.

### Non-traditional aspects of care

There are other potential targets of cancer that extend beyond the traditional boundaries of anti-cancer therapy. Calculating the personal risk for patient outcome comes into sharper focus, since it has been shown that there is a generational shift in metabolic risk factors [52], which will influence future generations in life expectancy, combined with co-morbidities and in quality in life (QL). A Cochrane analysis assessing the effects of psychosocial interventions for an improvement of QL revealed that it can be “*concluded, that nurse-delivered interventions comprising information combined with supportive attention may have a beneficial impact on mood in an undifferentiated population of newly diagnosed cancer patients*” [53]. Although assumed to exist for some time now no correlation was observed until recently that sympathetic activation, triggered by prolonged emotional stress affects overall survival, tumor incidence, shorter survival, and perhaps, increased recurrence of breast cancer and metastasis [54,55]. More recently it has been shown in a mouse model that the stimulatory effect of sympathetic activation on bone metastasis can be blocked with a beta-blocker or by inhibiting RANKL signaling in cancer cells. It follows that this might explain the reduced survival rate of breast cancer patients experiencing severe depression and serves as one potential focus point in the future to use beta-blockers as an adjuvant therapy for women with breast cancer [56]. Potential variables include other future scientific information, e.g., spiritual care [57] and their compilation with their biological targets.

#### Patient response

Despite these improvement, cancer patients more often present with locally advanced tumor categories (cT3/4) such that only about 30% of patients undergoing primary surgery will typically have microscopically R0 resections performed [58,59]. Therefore, a multi-modal approach has been under investigation for the past 40-plus years [60] with the aim of increasing the local-regional tumor control by an increase in the proportion of radical tumor resections where the boundaries of the tumor can be defined. Concepts of down-staging and increasing the ability to achieve negative tumor margins through the use of a multimodal therapy approach in a neoadjuvant setting have infiltrated the everyday care of cancer patients. Therefore, patient response criteria in evaluating the effect of anti-cancer therapy [61-63] need to be revisited [64]. These new criteria must include not just statistically significant overall survival rates of a few weeks/months or selecting few genetic markers, but must also consider findings in basic laboratory tests, histopathology, biochemistry, imaging, quality of the patient’s life post-treatment as well as psychosomatic areas. All of these truly define the care for the cancer patient, and require investigative changes implemented during multimodal treatment. Although we know that responders have better tumor biology, health outcomes, and a good prognosis, no significant therapeutic consequences or patient management trials using this biochemically focused, lesion-altering approach have been reported to date [64] with one exception [65].

#### Prevention

According to the National Cancer Institute, prevention is “*the reduction of cancer mortality via reduction in the incidence of cancer*” [66]. Both models of carcinogenesis, the one from Vogelstein and Kinzler [19,67] and the one from Hanahan and Weinberg [68], which have been mentioned above, include the influence of risk factors with negative influence and those with positive preventive effects. Accomplishments in clinical research have included looking at changes in lifestyle, reducing risk factors, preventing infections (which contribute to about 18% of all cancer causes) [69], improving dietary intake, as well as incorporating molecular biology. Only by understanding the complete pathway of carcinogenesis that includes variables such as biology and tumor metabolism, along with incorporating new ways of medical therapy (chemoprevention) modifying cancer-causing factors, and better identifying those with genetic predispositions for cancer will be able to make a dramatic leap forward in the evolving approaches to cancer. Early detection is an oft-used phrase which should be commonplace—buoyed by improvements in laboratory, radiographic, endoscopic tests and physical examinations. In reality, using biomarkers for the diagnosis of cancer in early tumor stages where a higher chance of cure is possible, or improved access to screening programs, is still lacking. Even one of the most widely used screening programs, PSA for prostate cancer, has failed, clearly illustrating the difference between the wishes of scientists and reality in the clinic [70].

Although a multi-billion ‘nutrition’ market has evolved with the primary aim of lessening exposure to cancer-promoting agents and improving host defense mechanisms, this has so far not provided evidence that this nutritional supplements lead to a decrease of the individual cancer risk [71]. Furthermore, it does not seem clear why this failure to greatly impact cancer rates occurs. Is this a dosing issue? Or is it that the nutrients themselves do not help? A large-scale study revealed an inverse relation between selenium intake, and reduction of prostate risk, as it was shown in a Dutch cohort trial [72].

Already in clinical use is a cancer prevention strategy: the elimination of precancerous lesions by colonoscopy for colorectal polyps, but its increasing use without a proven concept and leading to enormous health costs had recently been called into question [73].

### Summary

#### Joining forces

Widespread anti-cancer therapy may be more successfully accomplished for patients through joining forces of all the aspects discussed here. Combining a renewed focus on a cancer classification which includes not just histomorphological organ-specific variables as size, lymph node and/or distant tumor spread. We concede that this was necessary in the past and these significant achievements in anticancer therapy lead to standardization as well as they made it possible, that results had been comprehensible worldwide. This has been one major basis for the achievements in the War on Cancer. With the increased molecular and biocomputional information and understanding of all different cancer research disciplines, now might be the time to pivot to a combination of a personalized and an individualized diagnosis and treatment strategy. Such a personalized strategy could integrate all necessary different patient- and tumor-associated variables with the consecutive stratification of anticancer-treatment. Despite a straight forward orientated prevention strategy, the combination with patient- and tumor-associated variables in terms of response to anticancer treatment could result in the necessary modification of the stratified anticancer treatment. For that, such a strategy would be of enormous practical use to the clinician as well as for the research community, joining forces more effectively.

The effects of such a combined strategy (Figure 1) could be:

1. a tumor- *and* patient-orientated predictive cancer-staging system with the stratification of different forms of *personalized* therapy and

2. the development of a standardized multivariable response evaluation system with the consequence of future standardized therapy modifications (*individualized* cancer strategy).

”*Imaging A World Without Cancer*“ is clearly a vision. For its realization, a global personalized and individualized anticancer strategy could be fundamental as both could integrate patient- and tumor-associated achievements in research in an adoptable and cost sensitive manner.

### Support

The manuscript was supported by the Theodor-Billroth-Academy® (TBA®) and INCORE, (International Consortium of Research Excellence) of the (TBA®).

## Abbreviations

AJCC: American Joint Committee on Cancer; ASCO: American Society of Clinical Oncology; CCR: Colorectal carcinoma; CML: Chronic myelogenous leukaemia; QL: Quality in life; SNP: Single nucleotide polymorphisms; UICC: International Union Against Cancer.

## Competing interests

No author has something to disclose or competing existing interests.

## Authors’ contributions

This manuscript contains original material that has not been previously published. The content of the manuscript are discussions with each single author over a 10-year time period. All authors contributed in discussing the contents and approval of the manuscript.

## Authors’ information

BB, http://www.linkedin.com/in/bruecher

GL, http://www.linkedin.com/pub/gary-lyman/5/11a/a48

RH, http://www.linkedin.com/pub/richard-van-hillegersberg/24/ab3/a18

RP, http://surgery.osu.edu/oncology/directory/

FL, http://www.linkedin.com/pub/dir/Florian/Lordick

HKY, http://www.linkedin.com/pub/dir/Kwang/Yang

TU, http://www.linkedin.com/pub/toshikazu-ushijima/25/579/47

KGY, http://www.linkedin.com/pub/han-kwang-yang/51/39/7ba

TS, http://www.linkedin.com/pub/toshikazu-ushijima/25/579/47

WP, http://www.ihpba.org/69_Poland-National-Chapter.html

GW, http://www.wco.pl/esso2012/scientific-committee

VV, http://www.linkedin.com/pub/vic-verwaal/21/426/710/nl

AG, http://www.linkedin.com/pub/alfredo-garofalo/11/767//25

DU, http://www.linkedin.com/pub/domenico-domenico-genovesi/31/a68/85

FR, http://www.linkedin.com/pub/franco-roviello/20/b7b/382

HUS, http://de.wikipedia.org/wiki/Hans-Ulrich_Steinau

MP, http://www.linkedin.com/pub/mladjan-protic/b/81/967

TW, http://www.vitals.com/doctors/Dr_Timothy_J_Wallace.html

MD, http://de.linkedin.com/pub/martin-daumer/12/8b5//489

NM, http://www.linkedin.com/pub/nita-maihle/5/bb6/197

TJR, http://www.linkedin.com/in/thomasjreidiiimdphdfacp

MD, http://www.esmo.org/Membership/ESMO-in-Your-Country/National-Representatives/Michel-Ducreux-France

YK, http://www.linkedin.com/pub/dir/Yuko/Kitagawa

AK, http://www.cancerresearch.org/about/scientific-advisory-council/alexander-knuth

BZ, http://www.linkedin.com/pub/bruno-zilberstein/28/74a/791

SS, http://www.linkedin.com/pub/scott-steele/39/b21/15b

ISJ, http://www.linkedin.com/pub/ijaz-jamall-ph-d-dabt/1b/69/b92

## Pre-publication history

The pre-publication history for this paper can be accessed here:

http://www.biomedcentral.com/1471-2407/14/186/prepub
